# Network dynamics associated with experience-dependent plasticity in the rat somatosensory cortex

**DOI:** 10.1186/1471-2202-12-S1-P246

**Published:** 2011-07-18

**Authors:** Seif Eldawlatly, Karim Oweiss

**Affiliations:** 1 Electrical and Computer Engineering Dept., Michigan State University, East Lansing, MI 48824, USA; 2 Neuroscience Program, Michigan State University, East Lansing, MI 48824, USA

## 

Experience-dependent plasticity is hypothesized to occur in the rat barrel cortex as a result of whisker pairing induced by depriving all but two whiskers. A significant change in the response of individual neurons in the barrels of the spared whiskers is observed compared to their response before pairing. The over-usage of the spared whiskers results in an increased similarity in the sensory-evoked response of the neurons in the barrels of spared whiskers that may indicate an actual merging of the two barrels.

The goal of this study is to examine the application of Dynamic Bayesian Networks (DBNs) in tracking the network dynamics associated with this type of plasticity in layer V of the rat barrel cortex. DBN is an efficient graphical model that fits the observed spike train data by explaining the temporally precise spiking of each observed neuron while accounting for the spiking pattern of the entire observed population. We have recently demonstrated the effectiveness of DBN to infer stimulus-specific networks in the rat barrel cortex in response to the stimulation of individual whiskers. This form of network coding provides more information about the stimulus than both rate and temporal codes. In this study, we examined the changes that occur to these stimulus-specific networks as a result of whisker pairing. In 4 subjects, 32-channel multi-electrode arrays were chronically implanted to record from layer V of the barrel cortex. One week post implantation, sensory-evoked activity was recorded (control data) over a number of days after which all but two whiskers on one side of the rat’s mystacial pad were trimmed. Sensory-evoked activity was then recorded 1 to 2 days and 6 to 7 days post-trimming (plasticity data). Spike trains within 100 ms of stimulus onset of both control and plasticity data for each of the spared whiskers were then analyzed using DBN to infer stimulus-specific causal networks.

To assess plasticity at the individual neuron level, the number of evoked spikes by the neurons as well as the first-spike latency in response to the deflection of each of the spared whiskers were measured for both control and plasticity data. The difference in the number of evoked spikes and in the first-spike latency across the spared whiskers was found to decrease significantly 6 to 7 days post whisker trimming (Normalized difference in evoked spikes for control data: 0.41±0.23, 6-7 days post-trimming: 0.29±0.21; Normalized difference in first-spike latency for control data: 0.18±0.12, 6-7 days post-trimming: 0.15±0.11, *P* < 0.05, paired *t*-test). This indicates an increased similarity in the response of individual neurons to the spared whiskers as a result of the pairing. At the network level, a significant increase in the similarity between the stimulus-specific networks across the spared whiskers was observed that is proportional to the number of days of whisker pairing (Network similarity for control data: 0.58±0.1, 1-2 days post-trimming: 0.66±0.1, 6-7 days post-trimming: 0.71±0.1, *P* < 0.05, paired *t*-test). This finding suggests an experience-dependent plastic change in the neuronal networks of layer V that is consistent with Hebbian plasticity.

